# Pernicious Anæmia

**Published:** 1899-09

**Authors:** 


### PERNICIOUS ANÆMIA.

A fatal form of anæmia. It is an extreme anæmia advancing steadily, or with slight remissions, toward a fatal ending; yet no cause can be detected for the profound and disastrous alteration the blood is undergoing, nor, indeed, can any adequate cause be discerned for its origin. To pernicious anæmia belong, therefore, most of the cases of "essential" or "idiopathic anæmia," which since the time of Addison have been reported.

The disorder is most frequent in women, and has been especially observed in child-bearing women; still, it also often happens in men, especially before the age of forty. It sometimes seems to have its origin in long-continued dyspepsia or diarrhœa, or to arise after protracted hemorrhages or incessant worry—after indeed slowly but steadily acting debilitating influences; and it has been noted to be of parasitic origin. But in the majority of instances it originates seemingly without cause, and although it has periods of deceptive improvement which may last for months, or, as I have known, even for a year, it usually progresses relentlessly toward a fatal issue. It is true that recently some cases of recovery have been recorded by me under the blood treatment, but of these it is not quite certain that they presented all the well-marked and characteristic symptoms.

What are these characteristic symptoms? An insidious beginning for the obvious anæmia, except at times when this develops itself in the pregnant state; pale tongue; bloodless lips; pearly eye, becoming paler, more bloodless, more pearly, from week to week; breathlessness; palpitation of the heart, especially on exertion; weak digestion; constipation, or constipation alternating with diarrhœa; loud systolic murmurs in the heart, and venous hum in the jugulars; vertigo; finally, extreme exhaustion, sluggishness of mind, fainting fits and dropsy, without persistent albumen in the urine, or disease of the liver or enlargement or valvular disease of the heart, to account for it. In the later stages, too, hemorrhages from the nose and from the gums are not uncommon; and hemorrhages from the uterus or from the kidneys or into the skin and into the retina, may also be noticed; the latter especially is very frequent. Yet, notwithstanding all these grave signs, the body appears well nourished; there is certainly no decided emaciation, except in instances in which fever is more commonly marked. Now, fever is a significant feature of progressive pernicious anæmia; it has been present in every case that I have met with. It is not an early symptom, belonging to the full development or to the latter part of the disease. It is of very irregular type, and not of high intensity, the temperature rarely exceeding 103 degrees F. It is apt to be continued, or to show occasional exacerbations, followed by remissions, the febrile state lasting for days, or even for a week or two at a time; then there are periods of shorter or longer duration when it wholly disappears, to come on again in an outbreak attended with all the usual signs of a febrile paroxysm for which no cause is apparent. Toward the end of the case it is not unusual for the anæmic fever to have entirely ceased, and for the temperature to have fallen below the normal standard. The disease may run an acute course.

The state of the blood in this perilous malady has naturally been made a subject of minute investigation; the red globules are pale and strikingly diminished in number; the white corpuscles are not relatively altered, they may remain normal, and seem to be increased, because the red globules are much fewer. The paleness of the red corpuscles is due to deficiency of hæmaglobin. The shape of these was stated by Eichorst to be characteristically changed, in so far at least that the blood contains a quantity of ill-developed, small, spherical red corpuscles. But these are not pathognomonic. Nucleated red corpuscles were detected in the blood of all the patients examined by Howard; and a much larger proportion than is found normally of small disks of deep color is regarded as important.

Of the real cause of the disease we are in ignorance. No constant lesion of the blood-making glands has been found to explain the steady and destructive impoverishment of the blood. The structure of the spleen and of the lymphatic glands is not altered; the marrow of the bones may or may not be. The most constant lesion is fatty degeneration of the heart, often associated with the same change in the inner coat of the large arteries. A recent writer argues against the separation of pernicious anæmia from chlorosis.—From *Provisional Hand-Book of Hæmatherapy*, published by the Bovinine Company, whose advertisement appears in this number.

